# Supplementary material for: A casino in my pocket: Gratifications associated with obsessive and harmonious passion for mobile gambling
Source: PLoS One. 2021 Feb 24;16(2):e0246432. doi: 10.1371/journal.pone.0246432 (PMC7904185; doi:10.1371/journal.pone.0246432)
Supplement: S1 File — (DOCX) [file pone.0246432.s001.docx]

**Supplementary File 1**

**S1 Table. Survey constructs and items**

| *Construct* | *Item* |
| --- | --- |
| **Arousal** [1] | ARL1: I use mobile gambling apps because it raises my level of adrenaline. |
|  | ARL2: I use mobile gambling apps because it keeps me on the edge of my seat. |
|  | ARL3: I use mobile gambling apps because it stimulates my emotions. |
|  | ARL4: I use mobile gambling apps because it excites me. |
| **Entertainment** [2] | Ent1: I use mobile gambling apps because it’s entertaining |
|  | Ent2: I use mobile gambling apps because I just like to use it |
|  | Ent3: I use mobile gambling apps because it’s enjoyable |
| **Information Seeking** [2] | Info1: I use mobile gambling apps because it’s a new way to do research for bets |
|  | Info2: I use mobile gambling apps to get information for free |
|  | Info3: I use mobile gambling apps to look for information |
|  | Info4: I use mobile gambling apps because it’s a new way to do research for bets |
| **Pass Time** [2] | PsTm1: I use mobile gambling apps because it passes time when bored |
|  | PsTm2: I use mobile gambling apps when I have nothing better to do |
|  | PsTm3: I use mobile gambling apps to occupy my time |
| **Relaxation** [3] | Relax1: Using gambling apps allow me to unwind. |
|  | Relax2: Using gambling apps relaxes me |
|  | Relax3: Using gambling apps is a pleasant rest |
| **Social Interaction** [4] | Socl1: My friends and I use gambling apps as a reason to get together. |
|  | Socl2: Often, a group of friends and I will spend time using gambling apps. |
| **Harmonious Passion for Mobile Gambling**  [5] | HPass1: This gambling activity allows me to live memorable experiences. |
|  | HPass2: This gambling activity is in harmony with the other activities in my life. |
|  | HPass3: The new things that I discover with this gambling activity allow me to appreciate it even more. |
|  | HPass4: This gambling activity reflects the qualities I like about myself. |
|  | HPass5: This gambling activity allows me to live a variety of experiences |
| **Obsessive Passion for Mobile Gambling**  [5] | OPass1: I cannot live without this gambling activity. |
|  | OPass2: I am emotionally dependent on this gambling activity. |
|  | OPass3: I have a tough time controlling my need to play this gambling activity. |
|  | OPass4: I have almost an obsessive feeling for this gambling activity. |
|  | OPass5: The urge is so strong, I cannot help myself from playing this gambling activity. |
| **Problem Gambling Severity Index (PGSI)**  [6] | PGSI1: Have you bet more than you could really afford to lose? |
|  | PGSI2: Have you needed to gamble with larger amounts of money to get the same feeling of excitement? |
|  | PGSI3: Have you gone back on another day to try to win back the money you lost? |
|  | PGSI4: Have you borrowed money or sold anything to gamble? |
|  | PGSI5: Have you felt that you might have a problem with gambling? |
|  | PGSI6: Have people criticized your betting or told you that you had a gambling problem, whether or not you thought it was true? |
|  | PGSI7: Have you felt guilty about the way you gamble or what happens when you gamble? |
|  | PGSI8: Has gambling caused you any health problems, including stress or anxiety? |
|  | PGSI9: Has your gambling caused any financial problems for you or your household? |
| **General Mood**  [7] | Please reflect on the PAST MONTH of your life and rate the following emotions in terms of how frequently you experienced each (1 = not at all, 7 = extremely much).  Pleasant   - Happy - Joy - Pleased - Enjoyment/Fun - Glad - Delighted - Contented   Unpleasant   - Angry - Fear/anxiety - Frustrated - Depressed - Annoyed - Sad - Gloomy |

1. Sherry JL, Greenberg BS, Lucas K, Lachlan K. Video game uses and gratifications as predictors of use and game preference. Playing Video Games: Motives, Responses, and Consequences. 2006. doi:10.4324/9780203873700

2. Papacharissi Z, Rubin AM. Predictors of internet use. J Broadcast Electron Media. 2000;44: 175–196. doi:10.1207/s15506878jobem4402_2

3. Smock AD, Ellison NB, Lampe C, Wohn DY. Facebook as a toolkit: A uses and gratification approach to unbundling feature use. Comput Human Behav. 2011;27: 2322–2329. doi:10.1016/j.chb.2011.07.011

4. Stafford TF, Stafford MR, Schkade LL. Determining uses and gratifications for the internet. Decis Sci. 2004;20: 1–21. doi:10.1111/j.00117315.2004.02524.x

5. Rousseau FL, Vallerand RJ, Ratelle CF, Mageau GA, Provencher PJ. Passion and gambling: On the validation of the gambling passion scale (GPS). J Gambl Stud. 2002;18: 45–66. doi:10.1023/A:1014532229487

6. Ferris J, Wynne H. The Canadian Problem Gambling Index : Final report. Can Cent Subst Abus. 2001. doi:10.1007/s10899-010-9224-y

7. Diener E, Emmons RA. The independence of positive and negative affect. J Pers Soc Psychol. 1984;47: 1105–1117.
