# Supplementary material for: A casino in my pocket: Gratifications associated with obsessive and harmonious passion for mobile gambling
Source: PLoS One. 2021 Feb 24;16(2):e0246432. doi: 10.1371/journal.pone.0246432 (PMC7904185; doi:10.1371/journal.pone.0246432)
Supplement: Attachment — Submitted filename: PLoS One Response to Reviewers_FINAL.docx [file pone.0246432.s002.docx]

Response to Reviewers

| ***Reviewer Comment*** | ***Response*** |
| --- | --- |
| **R1.1** The authors state that “only a minority of users develop uncontrolled gambling habits associated to tangible negative outcomes.” This characterization suggests that diagnosed Gambling Disorders are the only real or ‘tangible’ instances of harm arising from gambling. Multiple bankruptcies, and enduring troubles with one’s credit rating are certainly tangible harms that can be done by excess gambling, but would not contribute to a diagnosis of Gambling Disorder. The recognition of a broader sphere of sub-clinical gambling-related harms is part of the emerging Public Health perspective on problem gambling (e.g. Wardle et al., 2019, BMJ). The authors should remove or rephrase this passage to accurately reflect the broader landscape of gambling problems. | The reviewer makes a fair point. We have now rephrased this statement in the revised version;  *Gambling harms can have severe adverse effects on individuals, families and society. However, for a subgroup of highly involved individuals, gambling can be considered a harmonious passion performed in a way in which it fulfills specific needs and promotes positive affect.*  We have also added an additional sentence in the introducing to reflect the broader impact of gambling problems;  *The prevalence of problem gambling is estimated to vary between 0.1% and 5.8% in 2019 in different countries around the globe [1]* |
| **R1.2** The authors suggest in this passage (and throughout the manuscript) that some patterns of highly-involved gambling are “adaptive” or “healthy”. I believe that this, too, is out of step with the available evidence, and beyond the scope of the authors’ obtained results. Large-scale longitudinal studies have clearly linked gambling involvement to gambling problems (Binde, Romild, & Volberg, 2017, IGS). The obtained results in this study do not show that harmonious passion is protective against gambling problems, but rather that it is not related to gambling problems (or the lack thereof). I believe that a more accurate characterization throughout this manuscript would say that harmonious passion could permit frequent gambling without elevating individuals’ existing risk of experiencing gambling problems. | The reviewer makes a fair point. Based on the available evidence base, it is more correct to refer to “problematic” versus “non-problematic” intensive involvement in gambling, than to “healthy” or “adaptive” gambling patterns. We have carefully revised the whole manuscript accordingly.  We have also revised the reference to high involvement in the abstract as follows:  *However, for a subgroup of highly involved individuals, gambling can be considered a harmonious passion that permits frequent gambling without elevating individual’s risks of experience problem gambling manifestations.* |
| **R1.3** The abstract appears to say there is only a small incidence of “tangible negative outcomes” associated with gambling involvement, but concludes with a suggestion to reduce harmful gambling by encouraging harmonious passion. Could the authors clarify the extent to which problem gambling is a serious concern in society? | Following this suggestion, we modified the last sentence of the abstract to state that gambling is an internationally relevant public health issue;  *Based on these findings, and given that problematic gambling is an internationally relevant public health issue (the prevalence of problem gambling is estimated to range from 0.1% to 5.8% in different countries), we suggest interventions focusing on specific uses and gratifications associated with an obsessive passion for mobile gambling may be effective in reducing problematic usage patterns*  In addition, we also added a sentence at the very beginning of the introduction to acknowledge it;  *Gambling is a popular and common pastime across many countries, yet the harms associated to gambling are nowadays recognized as an important public health issue [1]*  Finally, we also added a sentence in the introduction about the prevalence of problem gambling (see comment R1.1) |
| **R1.5** The authors suggest that interventions focusing on obsessive passion “would be most effective in reducing problematic usage patterns.” I am not sure that this is implied by the evidence presented. One would need a comparative study involving multiple intervention types to reasonably suggest that any one of them could be “the most effective”. Would the authors be comfortable saying such an intervention “may be effective”, rather than “would be most effective”? Introduction | We take this point on board. In the revised version, we now state;  *…we suggest interventions focusing on specific uses and gratifications associated with an obsessive passion for mobile gambling may be effective in reducing problematic usage patterns.* |
| **R1.6** For the reader’s convenience, on line 38, could the instructors stipulate roughly what percentage of the population experiences gambling problems (e.g. Potenza et al., 2019, Nature Reviews Disease Primers). | This sentence of the abstract has been modified as follows:  *Based on these findings, and given that problematic gambling is an internationally relevant public health issue (the prevalence of problem gambling is estimated to range from 0.1% to 5.8% in different countries), we suggest interventions focusing on specific uses and gratifications associated with an obsessive passion for mobile gambling may be effective in reducing problematic usage patterns*  We decided to mention the numbers obtained from Calado & Griffiths (2016) which are actually the numbers reported in the review by Potenza et al.  Calado, F. & Griffiths, M. D. Problem gambling worldwide: an update and systematic review of empirical research (2000–2015). *J. Behav. Addict.* **5**, 592–613 (2016). |
| **R1.7** On line 45, could the authors provide a citation to support the claim that “some people gamble frequently with a sense of volition, and without experiencing negative consequences such as financial problems”. I am not aware of any studies that specifically look at volition during gambling activities. | This dimension of volition is central in the DMP theory (and related scales). According to this request, three references were added on line 82 of the revised manuscript (line 45 of the original draft) i.e.  Rousseau FL, Vallerand RJ, Ratelle CF, Mageau GA, Provencher PJ. Passion and gambling: On the validation of the gambling passion scale (GPS). J Gambl Stud. 2002;18: 45–66. doi:10.1023/A:1014532229487  Morvannou A, Dufour M, Monson E, Roy É. Passion for gambling: a scoping review. Int Gambl Stud. 2018;18: 460–476. doi:10.1080/14459795.2018.1459777  Oikonomidis A, Palomäki J, Laakasuo M. Experience and Passion in Poker: Are There Well-Being Implications? J Gambl Stud. 2019;35: 731–742. doi:10.1007/s10899-018-9795-6 |
| **R1.8** On line 56, could the authors clarify what they mean by “sensors”? Coming from a slot machine research background, it is my view that modern slot machines can be highly sophisticated in many of the same ways as smartphones. Clarity is needed to understand what the authors mean when they say that smartphones afford unique gambling experiences. | We now provide more clarity on how smartphones provide unique gambling opportunities;  *Through an array of data collection opportunities provided by smartphones, mobile gambling apps are able to personalize gambling, in-play promotions for example, in a way traditional or even other forms of online gambling cannot. Gambling apps can be run on the background, prompting notifications of gambling opportunities. They can technically utilize data such as the users’ location, users’ list of contacts, their credit card balance, their use history among other things, to which even the most sophisticated modern slot machines do not have access to.* |
| **R1.9** On line 51, there may be a typo in the phrase “opportunities to gambling”. | This typo has now been corrected |
| **R1.10** On line 114, the authors compare gambling passion to sports, music, games, writing, work, and family. I wonder if it is appropriate to also compare gambling to other addictive substances and behaviours? Addictive things are unique in that they function to progressively diminish cognitive control, and so it seems reasonable to expect that passionate use of addictive things may naturally give way to uncontrolled, or ‘obsessive’ use patterns over time. Notably, this view seems to be contra-indicated by the authors’ non-significant correlations between gambling experience and both forms of passion. In any case, I am interested to know how the concept of passion could be applied to addictive things beyond gambling. Is it reasonable to say that there are passionate, high-frequency cocaine users, for whom drug use is both adaptive and healthy? | As Reviewer 1 is aware, problem gambling is akin to addictive behaviours and the available evidence base resulted in aligning gambling disorder to substance use disorder in the DSM-5.  Most drugs have devastating effects when intensively consumed, and to our knowledge few studies have applied the DMP to psychoactive substance. Yet, it was found that harmonious consumption of cannabis is positively associated to well-being. In order to take into account the comment made by the reviewer, we added the following sentence in our manuscript, in the section where we discuss the DMP in relation to the incentive-sensitization theory;  *Although the DMP has only rarely been used in the context of drug use, a study focusing on recreational marijuana consumption showed that harmonious passion toward its consumption is related to elevated life satisfaction and lower consumption-related negative consequences, whereas the opposite pattern is observed with regard to obsessive passion scores.*  Yet, and as already mentioned in relation to comment R1.2; we no longer use the terms “healthy” or “adaptive” when it comes to intensive (but unproblematic) gambling. |
| **R1.11** The authors’ definitions of harmonious and obsessive passion on line 122 stipulate that the key difference in harmonious passion is that the person “still remains in control” of their gambling behaviour. In some sense, it seems that the underlying construct of these forms of passion could be boiled down to whether or not gambling has become a habit, continuing irrespective of reward or punishment (e.g. Everitt & Robbins, 2016, Annual Review of Psychology). Reading this passage, I found myself frequently comparing these forms of passion to the constructs of “liking” versus “wanting” drugs (Robinson & Berridge, 1993, Brain Research Reviews). Could the authors clarify in this section the aspects of DMP that distinguish it from existing models of habit formation? | According to this comment, we have now added a new paragraph which explains the distinction between the dualistic model of passion and influential models in the addiction field (incentive-sensitization theory and dual process models)  *The DMP constructs of harmony and obsession present similarities with constructs included in influential addiction models such as the classic dichotomy between “liking” and “wanting” (operationalized in the incentive-sensitization theory [44]) or the distinction between “controlled use” and “impulsive use” (operationalized in dual-process models, see e.g. [45]. Yet, an important difference between the DMP and these models is that the latter essentially focus on neurobiological processes whereas the DMP is conceptualized within a broader psychological framework that consider a wider range of psychological processes (including self, identity, or personality-related processes).* |
| **R1.12** The methods stipulate that the PGSI was scored on a 4 point Likert scale from 0 to 3. However, the supplied OSF data repository shows that each PGSI item was scored on a 5-point Likert scale from 1 to 5. Did the authors use a non-standard scoring method for this instrument, or might there be some mistake in the data file? Does this impact the values reported in the tables? On a related note, readers would benefit from an accounting of the rate of problematic gambling (total PGSI score > 7) in the data set. The authors suggest several times that these results are applicable to problem gamblers, but that is not clear based on the available data, which only shows that there is significant positive skew on each of the nine PGSI items. To assess the applicability of these findings to problem gamblers, could the authors specifically state how many problem gamblers they tested? | This was indeed a mistake in the datafile uploaded to OSF. This has now been corrected. Responses were self-reported on the standard 4-point Likert scale [“Never” (0), “Sometimes” (1), “Most of the time” (2), “Almost always” (3)].  In terms of problem gamblers, 48% of respondents reported a PGSI score > 7. We now include this information in the revised paper (line 96).  We also now provide a link to the OSF dataset in the paper itself i.e. All items used in the online survey can be obtained from <https://osf.io/643ja/> |
| **R1.13** The authors stipulate that participants used apps like Bet365 and PaddyPower, but that most participants were American. Could the authors clarify the extent to which participants used these specific apps, or provide some indication of which apps were being used by participants in the study? | We have data on the gambling apps used and now include the 5 most popular in the revised paper i.e. Bet 365, PaddyPower, DraftKings, FOX Bet Sportsbook, 888 Sport. |
| **R1.14** The authors stated that the median participant gambled about $50 per week. Is that US dollars? Could they provide some indication of the range of expenditures that was reported in the sample (e.g. $5 – 1,500)? | This is US dollars. We now add in details of the range;  *The median amount gambled was US$50 per week with a minimum and maximum of US$5 and US$2,500 respectively.* |
| **R1.15** The layout for Table 1 is quite difficult to follow. Different demographic questionnaires are confusingly intermingled. Could the authors separate these different questionnaires visually (e.g. by introducing white space between them)? | We have now attempted to make table 1 easier to follow visually. |
| **R1.16** The results are comprehensive and thoughtfully presented. The authors have done a great job here! | Thank you for this feedback. |
| **R1.17** The authors note here that obsessive passion is associated with negative mood, and harmonious passion with positive mood. Is it possible that these effects are driven by the generally-positive framing of prompts in the harmonious passion scale, and the generally-negative framing of prompts in the obsessive passion scale? For example, the HPass questions cover topics like appreciation (#3), liking oneself (#4), and having memorable experiences (#1), whereas the OPass questions cover topics like emotional dependence (#2), obsessions (#4), and being unable to live without gambling (#1). In essence, is it possible that people who generally feel good are more apt to endorse HPass, while people who feel bad are more apt to endorse OPass? If so, could this be reflected in the limitations? | The reviewer makes an interesting point. We specifically gathered our survey data over two waves to avoiding priming responses to the dependent variables (e.g. mood) based on answers to the independent variables (e.g. harmonious and obsessive passion). That said, the reviewer’s point is valid and we now include this potential prompting as a limitation;  *While we found harmonious and obsessive passion to be associated with mood, this could possibly be due the generally-positive framing of prompts in the harmonious passion scale, and the generally-negative framing of prompts in the obsessive passion scale. However, to mitigate this potential bias, the current study employed a two-wave methodology that avoid priming effect on our dependent variables (i.e. mood and problematic gambling).* |
| **R1.18** On line 327, should “aim” read “aimed”? | Thanks for spotting this. It is now corrected. |
| **R1.19** The authors argue that “indicators such as frequency of gambling involvement should not be considered as [signs] of problematic gambling patterns” based on the obtained evidence. I do not believe that their findings negate the strong association between gambling involvement and gambling problems (Binde, Romild, & Volberg, 2017, IGS). Involvement may not – by itself – be a sufficient indicator of problem gambling, but it is clearly a valuable indicator of the likelihood that an individual may experience gambling problems: people who are uninvolved in gambling are less likely to experience gambling problems. I would like to recommend that the authors remove this passage. | We have now removed this passage from the revised version. |
| **R1.20** The discussion section is quite lengthy, and some parts may not be absolutely germane to the authors’ exploratory analyses. In particular, the supplementary analysis in which social interaction reportedly mediates pass-time gratification in affecting Obsessive Passion is somewhat surprising to me, as it does not seem immediately obvious that mobile gamblers seeking a pass-time would do so seeking social interaction in mind. I have some further reservations about exploratory mediation modelling as an unreliable indicator of causality (e.g. Keele, 2015, American Journal of Evaluation). The authors have done a good job to keep from implying undue causality in this model, but I still wonder if it represents a necessary element of the manuscript. | What we were attempting to achieve with the post hoc analysis was to provide readers with an additional explanation of why these particular results might have emerged. On reflection, to ensure only conclusions supported solidly by are our data analysis are included, we have removed this passage from the revised version. |
|  |  |
| **R2.1** The title is not really informative. Rather than antecedents and consequences of mobile gambling passion, perhaps a more accurate title would be something like characteristics associated with obsessive and harmonious passionate of mobile gambling? | Thanks for this suggestion. We have now changed the title to;  *“A Casino in my Pocket: Gratifications Associated with Obsessive and Harmonious Passion for Mobile Gambling”* |
| **R2.2** The authors do a fantastic job providing a rationale for the present research. I think the introduction could be strengthened if the authors provide a summary of motives related to gambling. In particular, it would be helpful if the authors speak to previous research that potentially speaks to motives that may be associated with positive aspects of gambling, rather than gambling disorder. The authors may wish to also examine the Positive Play Index to further bring in previous research that looks at potential predictors of positive gambling experiences. | We would like to thank the reviewer for his/her positive comment. In the revised introduction, we added a short section about gambling motives;  *Past research suggests that gambling motives play a pivotal role when it comes to account for problematic versus non-problematic gambling involvement [19–21]. For example, it has been shown that enhancement-related motives (e.g., feeling high) and coping-related motives (e.g., to relieve negative affect) are vulnerability factors for problematic gambling, which is not the case of social motives (e.g., meeting and sharing with people in the gambling context) [22].* |
| **R2.3** I am curious if there were any differences between the 86 participants who did not complete the follow-up and those who did. Did the authors consider using imputation rather than list-wise deletion, especially if there are significant differences in variables of interest in the two groups? | The 1st survey wave collected data on demographics, passions for mobile gambling, and the uses and gratification variables. We have now run ANOVA tests and can confirm there is no significant differences between the two groups. We now include a footnote in the revised paper to this effect;  *Footnote 1 - ANOVA tests confirmed there were no significant differences between the 86 non-respondents and the main sample in their responses to survey 1.*  Imputation would not be appropriate in this case as the group of 86 did not provide any data for any of the dependent variables. |
| **R2.4** Please provide alphas from the present sample. | Cronbach’s alpha’s for all constructs are provided in table 2. All constructs had a value higher than 0.7. |
| **R2.5** The authors spend a considerable time in the discussion regarding the social aspect of mobile gambling. Yet, is online gambling really social? I believe the authors do not have a measure of type of mobile gambling participants engaged in. For example, if a person was engaging in mobile slots, I would argue that is not really a social activity. | We believe online and mobile gambling have become social activities. As documented in McGee’s (2020) recent qualitative study of 32 gamblers, having ‘banter’ about the different bets friends have put on through dedicated WhatsApp groups has become a normalised part of gambling. Even though the group are physically distance, mobile technology affords them the opportunity to socialise through gambling. Below is one quote from a gambler from McGee’s study to illustrate this point;  *‘You should see our WhatsApp group with bets! Come the weekend, we all be throwing our bets in it. All the boys getting the sweat on together. Saturday is just pure football on the group - full of bets!’ Tom, 31.*  In relation to problem mobile gambling: Sport betting is the classical example of mobile gambling. In Johansen et al’s recent paper (which we now reference in our revised paper), they also allude to the social aspects of sports gambling;  *Sports betting is often undertaken in group (e.g., friends, relatives; Johansen et al., 2019), as a form of rituals as members of communities. Hence, young adults’ identity might be bound up with a sense of membership of a gambling community to which the individual values and wants to belong …*  *Johansen, A. B., Helland, P. F., Wennesland, D. K., Henden, E., & Brendryen, H. (2019). Exploring online problem gamblers’ motivation to change. Addictive Behaviors Reports, 10, 100187.* [*https://doi.org/10.1016/j.abrep.2019.100187*](https://doi.org/10.1016/j.abrep.2019.100187)  However, the reviewer’s point is valid. Some mobile gambling activities are more social than others. In the revised paper, we now suggest that a comparison of social gratifications of different mobile gambling activities as an interesting area of future research;  *However, some mobile gambling activities such as sports betting are more likely to facilitate social interaction over others like mobile slots [78]. Future research should determine if the relationship between social gratification and problem gambling is stronger for some mobile gambling activities than others.* |
| **R2.6** In limitations, I would not that UGs assessed were from mobile games. There are key differences in mobile games and mobile gambling, and thus some UGs for mobile gaming may not apply to mobile gambling. | Thanks for this suggest and we now add this in as a limitation;  *Theory-wise, we operationalized six prominent U&Gs as antecedents of harmonious and obsessive passion. These particular U&Gs were adapted from studies of mobile gaming and may not be the most appropriate for mobile gambling. Future work could expand this analysis by developing U&G measures specific to the mobile gambling context. For example, U&Gs such as coping-escapism and nostalgia may be fruitful in explaining the outcomes of mobile gambling.* |
